# Targeted LC-MS/MS profiling of bile acids reveals primary/secondary bile acid ratio as a novel biomarker for necrotizing enterocolitis

**DOI:** 10.1007/s00216-023-05017-7

**Published:** 2023-11-08

**Authors:** Tingting Gao, Shaohua Hu, Weijue Xu, Zhiru Wang, Ting Guo, Feng Chen, Yingxuan Ma, Linlin Zhu, Faling Chen, Xiaomei Wang, Jin Zhou, Zhibao Lv, Li Lu

**Affiliations:** 1 Department of General Surgery, Shanghai Children’s Hospital, School of Medicine, Shanghai Jiao Tong University, Shanghai, China; 2 Department of Clinical Laboratory, Shanghai Children’s Hospital, School of Medicine, Shanghai Jiao Tong University, Shanghai, China; 3 Agricultural Information Institute, Chinese Academy of Agricultural Sciences, Beijing, China

**Keywords:** Bile acid metabolism, Primary/secondary bile acid ratio, Premature infants, Necrotizing enterocolitis

## Abstract

Bile acids (BAs) are involved in the development of necrotizing enterocolitis (NEC), which mainly occurs in preterm infants. We aim to identify the change of BAs in preterm infants and validate its potential value in the detection of NEC. Targeted liquid chromatography–tandem mass spectrometry (LC-MS/MS) was performed to measure the plasma BAs in healthy preterm infants and patients with NEC. By analyzing the level of BAs in healthy preterm infants, we found that the plasma concentrations of BAs were related to sex, gestational/postnatal age, birth weight, mode of birth, and feeding type after birth. The plasma levels of TCA, GCA, TCDCA, GCDCA, primary BAs, and total BAs and the primary/secondary BA ratio were decreased, while DCA, UDCA, and secondary BAs were increased in NEC. The primary/secondary BA ratio (cutoff point 62.9) can effectively differentiate NEC from healthy preterm infants, with an AUC of 0.9, a sensitivity of 94.5%, and a specificity of 78.1%. Combining the ratio with high-risk factors of NEC can better distinguish between NEC and control, with an AUC of 0.95. Importantly, significantly lower levels of primary/secondary BA ratio were found in infants with surgical NEC than in nonsurgical NEC cases. The cutoff point of 28.7 identified surgical NEC from nonsurgical NEC with sensitivity and specificity of 76.9% and 100%. Thus, our study identified that the primary/secondary BA ratio in the plasma can differentiate NEC from healthy preterm infants and effectively differentiate the surgical NEC from nonsurgical NEC. Therefore, LC-MS/MS was expected to be a novel measurement platform used to distinguish infants who are most in need of close monitoring or early surgical intervention.

## Introduction

NEC is one of the most common gastrointestinal emergencies in newborns, especially premature infants. Its incidence rate is between 3 and 15%, with a mortality rate ranging from 15 to 30%, and the mortality is as high as 100% in those with extensive full-thickness necrosis of the gut [1, 2]. Sex, gestational age, birth weight, mode of birth, feeding type, and intestinal bacterial colonization are all involved in NEC [3].

BAs include primary and secondary BAs. Cholic acid (CA) and chenodeoxycholic acid (CDCA), which are primary BAs, are synthesized in the liver, and conjugated with glycine or taurine to form binding primary BAs, including taurocholic acid (TCA), glycocholic acid (GCA), taurochenodeoxycholate (TCDCA), and glycochenodeoxycholate (GCDCA). Binding primary BAs are hydrolyzed, dehydroxylated, and transformed into secondary BAs by intestinal bacteria [4]. The secondary BAs mainly includes deoxycholic acid (DCA), lithocholic acid (LCA), ursodeoxycholic acid (UDCA), taurolithocholate (TLCA), glycolithocholate (GLCA), taurodeoxycholate (TDCA), glycodeoxycholic acid (GDCA), tauroursodeoxycholic acid (TUDCA), and glycoursodeoxycholic acid (GUDCA). Primary and secondary BAs function through the BAs receptors, such as FXR, VDR, TGR5, PXR, and RORγt [5]. Abnormal metabolism of BAs and expression of their receptors are related to various diseases [6–8]. The clinical data show that the colonization by *Lactobacillus* and *Enterococcus* is significantly delayed in children with NEC compared with healthy infants [9–12], which is negatively correlated with secondary BAs concentrations and positively correlated with primary BAs concentrations [13, 14]. Moreover, a recent study suggested that the coefficient of variation (CV) of total BAs in the fecal was a biomarker of NEC and could be developed as the first predictive test for NEC [15]. Based on these existing research results, we believe that BAs undergo changes in NEC and may be a biomarker for NEC.

The diagnosis of NEC is based on the clinical symptoms and characteristic radiological examination, which are defined using the modified Bell’s staging criteria [16]. Laboratory findings from the blood in patients with NEC include increased C-reactive protein (CRP) and serum amyloid A (SAA), abnormal white blood cell (WBC) count, thrombocytopenia, metabolic acidosis, hypoglycaemia or hyperglycaemia, and electrolyte imbalance, but all these indices are often nonspecific [17–19]. Thus, many studies have searched for new biomarkers for NEC in the blood. At present, the development of new diagnostic markers in blood mainly relies on detecting proteins in plasma, but these indicators face the problem of no standardized testing platforms. LC-MS/MS is routinely applied in the analysis of therapeutic drug monitoring, endocrinology including newborn screening, and toxicology in the clinic [20]. In recent years, LC-MS/MS for BAs profiling was developed and validated, which has demonstrated good application value in the diagnosis of diseases, including pediatric liver and intestinal diseases [21, 22]. However, there has been limited research on whether LC-MS/MS for detecting BAs can be used to find diagnostic biomarkers for NEC.

In this study, we characterized the factors influencing BAs metabolism in premature infants and investigated the diagnostic value of BAs in patients with NEC. The results suggested that BAs circulation in preterm infants was altered according to sex, gestational age/postnatal age, birth weight, mode of birth, and feeding type, which are almost all influencing factors of NEC. Importantly, further analysis suggested that a low level of primary/secondary BA ratio contributes to the clinical detection of NEC and surgical NEC.

## Materials and methods

### Sample collection and study participants

In our study, blood samples from preterm infants from Shanghai Children’s Hospital were collected between January 2020 and January 2022. This study was approved by the Ethics Committee of Shanghai Children’s Hospital (code number: 2019R083-E01). The inclusion criteria were as follows: healthy premature infants with gestational age <37 weeks. The exclusion criteria were as follows: (a) congenital intestinal malformations and other serious malformations, such as fetal gastroschisis and intestinal atresia; (b) spontaneous intestinal perforation; (c) incomplete clinical data or failure to follow the procedures to store samples. The diagnosis of NEC was based on the clinical symptoms and imaging examination, which were defined under the modified Bell’s staging criteria [16]. Plasma was isolated from blood samples immediately after collection. The plasma samples were stored at −80°C until use. The demographic characteristics including sex, gestational/postnatal age, mode of birth, birth weight, feeding type, Apgar score, and fecal occult blood were collected.

The blood of 32 premature infants collected at the onset of NEC (Bell stage ≥II) was enrolled in the study. For the control group, 128 healthy preterm infants were selected with pair match analysis (4:1) based on the gestational age, postnatal age, and birth weight. The healthy preterm infants were divided into four groups based on gestational age: infants aged <28 weeks, infants aged between 28 and 32 weeks, infants aged between 32 and 35 weeks, and infants aged >35 weeks; three groups by birth weight: infants <1000 g, infants between 1000 and 1500 g, and infants >1500 g; three groups by feeding type: breastfeeding, formula, and combination. The classification of Apgar score was as follows: 8–10, normal; 4–7, mild asphyxia; and 0–3, severe asphyxia. Postnatal age was grouped into weekly intervals.

### Sample preparation for LC-MS/MS detection

LC-MS/MS is applied in a multitude of important diagnostic niches of laboratory medicine. The technique has been routinely used in the identification of BAs [20–22]. Reagents, deuterium-labeled internal standards, standard products, and quality control products were purchased from Guangzhou ClinMeta Medical Device Co., Ltd. To run the assay, 100 μL of plasma (or standard products or quality control products) and 500 μL of ice-cold methanol with partial internal standards were vortexed vigorously for 5 min. After centrifugation at 13,000 rpm for 10 min, 400 μL of the supernatant was placed into autosampler vials and purged with nitrogen which was heated to 60 °C. After nitrogen purging, 100 μL of the dilute solution was added to vials and vortexed vigorously for 5 min. This vial was stirred well, and 5 μL of the solution was injected into the XBridge-T3 column (3.5 μm, 3.0 mm × 50 mm). Samples were prepared at room temperature.

### LC-MS/MS analysis

**Fig. 1:**
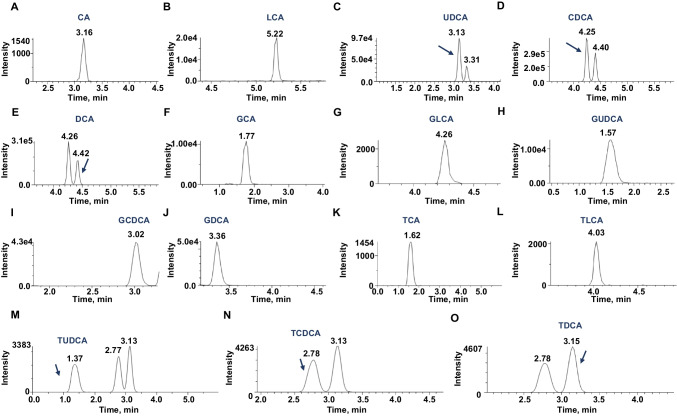
Representative chromatographic run of 15 BAs with mass transitions. Representative chromatograms for CA **A**, LCA **B**, UDCA **C**, CDCA **D**, DCA **E**, GCA **F**, GLCA **G**, GUDCA **H**, GCDCA **I**, GDCA **J**, TCA **K**, TLCA **L**, TUDCA **M**, TCDCA **N**, TDCA **O**

All plasma BAs were analyzed by LC-MS/MS (AB Sciex API3200MD and Shimadzu 20AD) in the clinical laboratory of Shuguang Hospital Affiliated to Shanghai University of Traditional Chinese Medicine by dedicated laboratory staff. LC-MS/MS was operated in multiple reaction monitoring (MRM) negative-ion mode to determine the concentration of all BAs. Analytical conditions are listed in Table 1. Samples were injected into the XBridge-T3 column (3.5 μm, 3.0 mm × 50 mm) at a flow rate of 500 μL/min, with an initial mobile phase composition of 90:10 (v/v) A: B for 0.5 min. Then, mobile phase composition was changed linearly to 40:60 (v/v) A: B over 5.5 min. Last, the column was washed with 10:90 (v/v) A:B for 2 min and re-equilibrated for 2 min with 90:10 (v/v) A:B before the next injection. The total run time between injections is 10 min. The optimized MRM transitions are summarized in Table 2, and the representative chromatographic run showing BA separation is shown in Fig. 1. The data were collected and processed using the instrument software Analyst 1.6.3. Calibration curves were calculated using weighted linear regression. The acceptance criteria of calibration curves are the correlation coefficient (*R*^2^) value of calibration curves should be at least 0.99 for all BAs. All QCs should be ±15% of nominal.

**Table 1:** Analytical conditions

Mass spectrometer	AB Sciex API3200MD
Software	Analyst 1.6.3
Ionization	Electrospray Ionization (ESI), negative
Curtain gas (CUR)	30 PSI
IonSpray voltage (IS)	−4500 V
Temperature (TEM)	600 °C
Ion source gas 1 (GS1)	50 PSI
Ion source gas 2 (GS2)	50 PSI
HPLC	Shimadzu 20AD
Mobile phase A	H_2_O
Mobile phase B	Methanol
Analytical column	XBridge-T3 column (3.5 μm, 3.0 mm × 50 mm)
Flow rate	500 μL/min
Injection volume	5 μL
Cooler temperature	15 °C
Column oven	40 °C

**Table 2:** Compound specific parameters for each bile acid species

Bile acids	Q1 (amu)	Q3 (amu)	Declustering potential (DP)	Collision energy (CE)	Entrance potential (EP)	Collision cell exit potential (CXP)	Internal standard (IS)	Retention time (RT) (min)
Cholic acid (CA)	407.2	343.2	−80	−22	10	8	CA-d5	3.16
Lithocholic acid (LCA)	375.2	375.2	−75	−22	10	8	LCA-d4	5.22
Ursodeoxycholic acid (UDCA)	391.2	391.2	−90	−20	10	12	DCA-d4	3.13
Chenodeoxycholic acid (CDCA)	391.2	391.2	−90	−19	10	4	DCA-d4	4.25
Deoxycholic acid (DCA)	391.2	391.2	−70	−20	10	8	DCA-d4	4.42
Glycocholic acid (GCA)	464.2	74.0	−95	−65	10	8	GCA-d4	1.77
Glycolithocholate (GLCA)	432.2	74.0	−80	−65	10	8	GLCA-d4	4.26
Glycoursodeoxycholic acid (GUDCA)	448.2	74.0	−90	−65	10	8	DCA-d4	1.57
Glycochenodeoxycholate (GCDCA)	448.2	74.0	−90	−60	10	10	DCA-d4	3.02
Glycodeoxycholic acid (GDCA)	448.2	74.0	−90	−65	10	5	DCA-d4	3.36
Taurocholic acid (TCA)	514.2	80.0	−128	−106	10	8	TCA-d4	1.62
Taurolithocholate (TLCA)	482.2	80.0	−102	−103	10	8	TLCA-d5	4.03
Taurochenodeoxycholate (TCDCA)	498.2	80.0	−115	−118	10	12	TCDCA-d5	2.78
Tauroursodeoxycholic acid (TUDCA)	498.2	80.0	−115	−118	10	10	TUDCA-d5	1.37
Taurodeoxycholate (TDCA)	498.2	80.0	−115	−118	10	6	DCA-d4	3.15
d4-Lithocholic acid (LCA-d4)	379.3	379.3	−110	−21	10	8	/	
d4-Deoxycholic acid (DCA-d4)	395.0	395.0	−130	−20	10	8	/	
d5-Cholic acid (CA-d5)	412.4	347.0	−120	−18	10	8	/	
d4-Tauroc (TCA-d4)	518.0	80.0	−125	−122	10	8	/	
d4-Glycocholic acid (GCA-d4)	468.0	468.0	−80	−65	10	8	/	
d4-Glycolithocholate (GLCA-d4)	436.4	74.2	−80	−65	10	8	/	
d5-Taurolithocholate (TLCA-d5)	487.2	80.0	−102	−103	10	8	/	
d5-Taurochenodeoxycholate (TCDCA-d5)	503.5	80.0	−115	−118	10	8	/	
d5-Tauroursodeoxycholic acid (TUDCA-d5)	503.4	80.0	−115	−118	10	8	/

### Statistical analysis

SPSS Statistics 23.0 software (IBM SPSS, NY, USA) was used for data collation and analysis. The statistical analysis was graphed using GraphPad Prism version 7 (GraphPad Software, CA, USA). Quantitative variables are expressed as medians (interquartile ranges, IQRs) and were compared by the Mann-Whitney *U* test. Qualitative variables are expressed as percentages and were compared by the chi-square test. One-way analysis of variance was performed for multiple sets of data.* P* <0.05 indicated a statistically significant difference. We evaluated the diagnostic performance of BAs using the receiver-operating characteristic (ROC) curve. A nomogram was employed that incorporated BAs and clinical characteristics, which we used for diagnosing NEC.

## Results

### Clinical characteristics of the participants

A total of 128 healthy preterm infants and 32 patients with NEC participated in the study. The clinical characteristics of the participants are shown in Table 3. There was no significant difference in sex, gestational age, mode of birth, birth weight, postnatal age, or Apgar score between the control and NEC groups. The breastfeeding rate was lower, and the incidence of fecal occult blood was higher in patients with NEC than in healthy preterm infants.

**Table 3:** General characteristics of the study participants

Characteristic		Value of the following group		*X*^2^	*P*
		CON (*n*=128)	NEC (*n*=32)		
Gender, *n* (%)	Male	73 (57.0%)	23 (71.9%)	2.350	0.159
	Female	55 (43.0%)	9 (28.1%)		
Gestational age (D), median (IQR)		214 (207 to 225)	215.5 (207.3 to 236.8)		0.252
Mode of birth, *n* (%)	Natural labor	33 (25.8%)	11 (34.4%)	0.948	0.377
	Caesarean	95 (74.2%)	21 (65.6%)		
Birth weight (g), median (IQR)		1410 (1234 to 1585)	1500 (1200 to 1919)		0.126
Postnatal age (D), median (IQR)		18 (8.25 to 27.75)	18 (8.25 to 32.5)		0.211
Feeding type, *n* (%)	Breastfeeding	82 (64.1%)	14 (43.8%)	17.533	<0.001
	Formula	9 (7.0%)	11 (34.4%)		
	Combination	37 (28.9%)	7 (21.8%)		
Apgar score, median (IQR)	1 min	8 (7 to 9)	8 (6.25 to 9)		0.507
	5 min	9 (8 to 9)	9 (8 to 9)		0.858
Fecal occult blood, *n* (%)	Positive	6 (4.7%)	17 (53.1%)	48.797	<0.001
	Negative	122 (95.3%)	15 (46.9%)		

### Postnatal change of BAs metabolism in premature infants

**Fig. 2:**
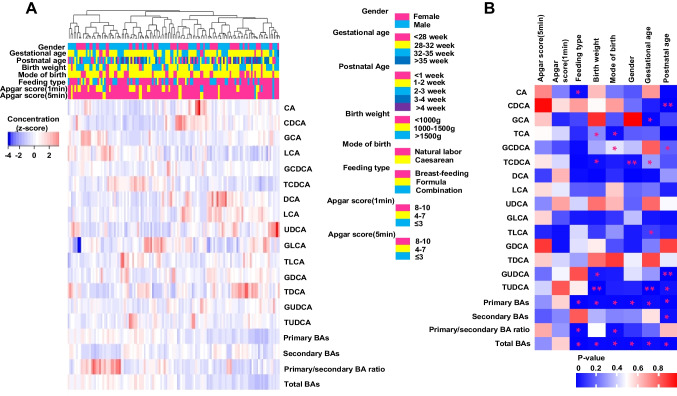
Correlation analysis between BAs levels and clinical parameters. **A** Plasma BAs levels in healthy preterm infants are shown according to clinical characteristics (gender, gestational age, mode of birth, birth weight, feeding type, postnatal age, and Apgar score). The primary BAs concentration is the sum of CA, CDCA, GCA, TCA, GCDCA, and TCDCA. The secondary BAs concentration is the sum of DCA, LCA, UDCA, GLCA, TLCA, GDCA, TDCA, GUDCA, and TUDCA. Total BAs are the sum of primary and secondary BAs. **B** BAs concentrations were compared under different characteristics. *P<0.05; **P<0.01; ***P<0.001

To investigate the change of BAs in the premature infants after birth, we first analyzed the relationships between BAs levels and important clinical indicators using the samples of healthy premature infants. The involved clinical characteristics, including sex, gestational/postnatal age, mode of birth, birth weight, feeding type, and Apgar score (1 and 5 min), were almost all influencing factors of NEC (Fig. 2A). As shown in Fig. 2B, the primary BAs were more susceptible to the change of clinical characteristics compared to secondary BAs. The total primary BAs and total BAs concentrations were both correlated with all the investigated clinical characteristics except for Apgar score, while the total secondary BAs concentrations were only correlated with postnatal age. Meanwhile, the ratio of total primary to secondary BAs concentrations was correlated with feeding type and mode of birth. All these results indicated that the primary BAs were correlated with the birth status and postnatal age, while the secondary BAs were mainly age dependent.

### Plasma BAs profile is significantly altered in infants with NEC

**Fig. 3:**
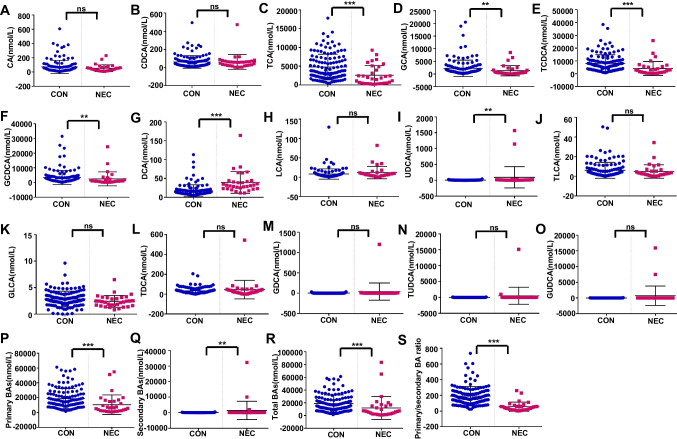
Comparison of plasma BAs levels between NEC patients and controls. **A** CA, **B** CDCA, **C** TCA, **D** GCA, **E** TCDCA, **F** GCDCA, **G** DCA, **H** LCA, **I** UDCA, **J** TLCA, **K** GLCA, **L** TDCA, **M** GDCA, **N** TUDCA, **O** GUDCA, **P** primary BAs, **Q** secondary BAs, **R** total BAs, and **S** primary/secondary BA ratio were compared between control (*n*=128) and NEC groups (*n*=32). *P<0.05; **P<0.01; ***P<0.001

Next, we compared the plasma levels of BAs in healthy preterm infants and patients with NEC. No differences were observed in the level of CA, CDCA, LCA, TLCA, GLCA, TDCA, GDCA, TUDCA, or GUDCA between the two groups (Fig. 3A, B, H, and J–O). The concentrations of TCA, GCA, TCDCA, and GCDCA were lower in patients with NEC than in healthy preterm infants (Fig. 3C–F), while the plasma levels of DCA and UDCA were higher in patients with NEC (Fig. 3G, I). The levels of primary BAs and total BAs and the primary/secondary BA ratio were decreased, while the levels of secondary BAs were increased in the patients with NEC compared with the healthy preterm infants (Fig. 3P–S). In summary, BAs metabolism was significantly altered in patients with NEC.

### Stratified analysis of differential BAs in NEC with different feeding types

**Fig. 4:**
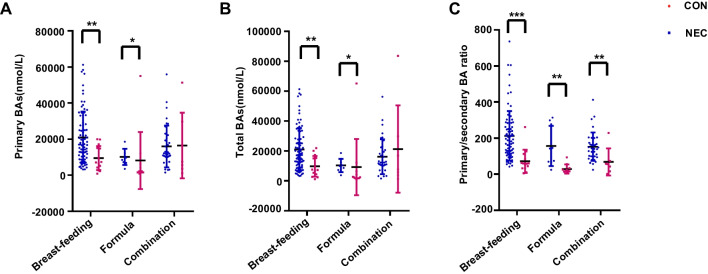
Hierarchical analysis of BAs based on different feeding types. **A** Primary BAs, **B** total BAs, and **C** primary/secondary BA ratio in NEC (*n*=32) and healthy preterm (*n*=128) were compared between the three feeding types. *P<0.05; **P<0.01; ***P<0.001

To study the diagnostic value of differential indicators in NEC, we first conducted a hierarchical analysis of the differential BAs affected by feeding type, including primary BAs, total BAs, and primary/secondary BA ratio. The results showed that the difference in primary and total BAs between the two groups is related to feeding type (Fig. 4A, B). Only the primary/secondary BA ratio was statistically significantly differed in the whole patient population with three different feeding types (Fig. 4C).

### Utilities of primary/secondary BA biomarkers for identifying NEC

**Fig. 5:**
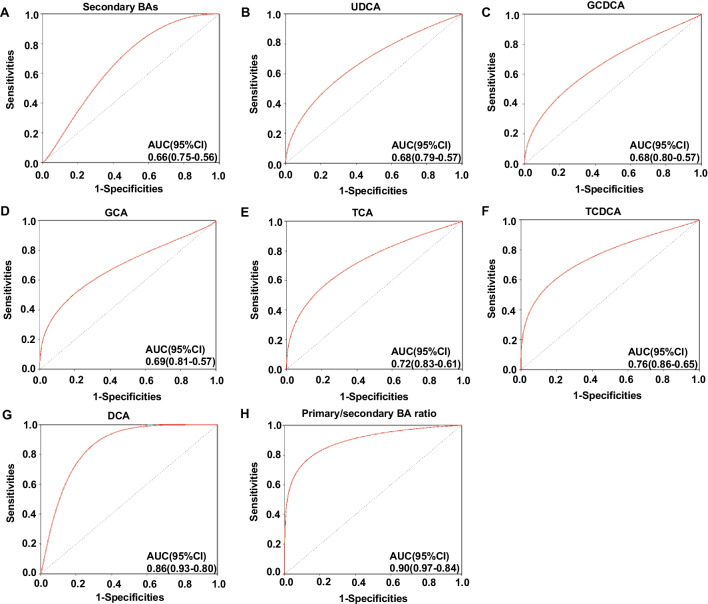
ROC curves to analyze the diagnostic value of differential BAs. **A** Secondary BAs, **B** UDCA, **C** GCDCA, **D** GCA, **E** TCA, **F** TCDCA, **G** DCA, and **H** primary/secondary BA ratio

Therefore, differential BAs not affected by feeding type, namely, GCA, TCA, DCA, UDCA, GCDCA, and TCDCA, as well as secondary BAs, and the primary/secondary BA ratio were chosen to evaluate their diagnostic value for NEC. The diagnostic value of secondary BAs, UDCA, GCDCA, and GCA was low with AUC of 0.66 (95% CI, 0.56–0.75), 0.68 (95% CI, 0.57–0.79), 0.68 (95% CI, 0.57–0.80), and 0.69 (95% CI, 0.57–0.81), respectively (Fig. 5A–D). The diagnostic value of TCA, TCDCA, and DCA was general with AUC of 0.72 (95% CI, 0.61–0.83), 0.76 (95% CI, 0.65–0.86), and 0.86 (95% CI, 0.80–0.93), respectively (Fig. 5E–G). The diagnostic value of the primary/secondary BA ratio was the best with an AUC of 0.90 (95% CI, 0.84–0.97) (Fig. 5H). The cutoff point of the primary/secondary BA ratio was 62.9 with a sensitivity and specificity of 94.5% and 78.1%, respectively, indicating a meaningful diagnostic value for NEC detection.

**Fig. 6:**
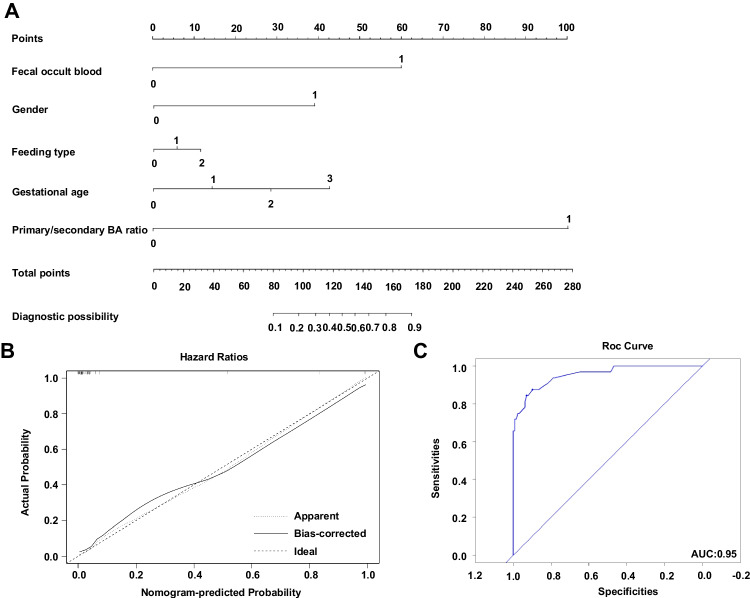
Nomogram constructed for NEC diagnosis. **A** Nomogram for NEC developed from the 160 preterm. The combination of fecal occult blood negative, female sex, gestational age (<28 w), breastfeeding, and primary/secondary BA ratio ≤ 62.9 was assigned a nomogram score of 0; fecal occult blood positive, male sex, gestational age (28–32 w), combinational feeding, and primary/secondary BA ratio >62.9 was assigned 1; formula feeding and gestational age (32–35 w) was assigned 2; and gestational age (>35 w) was assigned 3. **B** The calibration curve of the nomogram. **C** The ROC of the nomogram for NEC risk in the whole cohort

Next, we analyzed the ability of the primary/secondary BA ratio to assist in the clinical diagnosis of NEC in all 160 participants. The clinical characteristics for inclusion in the model were selected from among traditional influencing factors for NEC, including gender, gestational age, mode of birth, birth weight, feeding type, and fecal occult blood. The nomogram was constructed based on these risk factors and primary/secondary BA ratio (Fig. 6A). The calibration plot for the probability of NEC showed optimal agreement between the prediction by the nomogram and the actual observation (Fig. 6B). The AUC was calculated to evaluate the performance of the proposed nomogram, and the results showed that the combination of the primary/secondary BA ratio with clinical characteristics, such as the gender, gestational age, feeding type, and fecal occult blood, significantly improved the stratification of patients with NEC vs. healthy infants, with an AUC of 0.95 (Fig. 6C). These results confirm that the primary/secondary BA ratio might be a good diagnostic index for NEC.

### Primary/secondary BA ratio identified surgical NEC from nonsurgical NEC

**Fig. 7:**
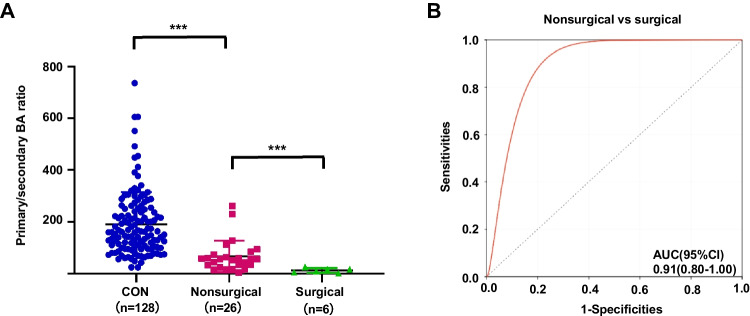
Comparison and ROC analysis of primary/secondary BA ratio between surgical and nonsurgical NEC cases. **A** The level of primary/secondary BA ratio in surgical NEC (*n*=6) was significantly decreased than that in nonsurgical cases (*n*=26). **B** Diagnostic ability of primary/secondary BA ratio to distinguish surgical NEC in the NEC group

Further analysis was performed between surgical NEC and nonsurgical NEC to validate the potential value of primary/secondary BA ratio in identifying surgical NEC from nonsurgical NEC. Significantly lower plasma level of primary/secondary BA ratio was observed in patients with surgical NEC than in nonsurgical cases (Fig. 7A). The ability of primary/secondary BA ratio in stratifying patients with surgical NEC vs. nonsurgical NEC was well with an AUC of 0.91 (Fig. 7B). Otherwise, using the plasma concentrations (28.7) of primary/secondary BA ratio in the NEC group as cutoff values for identifying surgical NEC from nonsurgical NEC cases had specificities of 100% and sensitivities of 76.9%. Thus, the primary/secondary BA ratio is a biomarker for diagnosing surgical NEC.

## Discussion

NEC is one of the most common gastrointestinal emergencies in newborn and is a major cause of morbidity and mortality in premature infants [23]. Studies have reported that some risk factors of NEC were responsible for the onset of NEC, such as gender, gestational age, mode of birth, birth weight, feeding type, and Apgar score [24]. In the current study, we found that the total BAs concentration and total of primary BAs concentration in plasma were correlated with all the explored clinical characteristics except for Apgar score, while total secondary BAs concentration was only affected by postnatal age. Thus, the primary BAs were affected by the birth characteristics and postnatal age, while the secondary BAs were age dependent. This is consistent with the conclusion that the establishment of an intestinal microbial flora is necessary for intestinal secondary BAs transformation and this process requires some months [25].

BAs are the end-products synthesized by the liver from cholesterol and are involved in liver, biliary, and intestinal diseases [26]. Plasma BAs can be used as diagnostic biomarkers to distinguish patients with schizophrenia or diabetes from healthy controls [27, 28]. Total BAs may help determine the etiology of acute pancreatitis in its early phase [29], while TUDCA-PSR and GUDCA-PSR have been used to discriminate intrahepatic cholangiocarcinoma from hepatocellular carcinoma, liver cirrhosis, and healthy controls [30]. In this study, we evaluated the concentrations of 15 BAs using LC-MS/MS and significant changes in BAs metabolism were found in NEC. Our results indicated that the primary/secondary BA ratio might be used as an additional useful indicator for NEC, especially surgical NEC. Early detection and proper treatment of NEC are key factors in improving its prognosis [31], and we will further investigate and estimate the value of the primary/secondary BA ratio in predicting NEC or differentiating NEC from feeding intolerance using larger sample size. Delaying surgical intervention may result in a higher morbidity and mortality. It is important to accurately identify surgical NEC cases at the onset of clinical presentation. Verified with a larger sample size, the detection of primary/secondary BA ratio may help doctors more accurately determine the timing of surgery in the future. Otherwise, LC-MS/MS is considered the gold standard for analysis of the BAs profile in serum samples [32]. In China, a standardized platform for the detection of BAs in the clinic has been built and authorized by the government, which will accelerate the clinical transformation rate of BAs-related indicators for NEC clinical diagnosis.

Long-term intravenous nutrition is an important risk factor for cholestasis, which could lead to abnormal BAs levels. To analyze the impact of this factor in our research, we analyzed the intravenous nutrition status of the enrolled samples and found that the intravenous nutrition time (from admission to blood sample collection) of the control and NEC groups did not exceed 10 days. Due to multiple studies showing that intravenous nutrition requires longer time to cause cholestasis [33, 34], we have not placed this indicator as an important consideration in our study. Additionally, we need to mention that the changes in BAs are very rapid, and we know we need to consider the effect of feeding on the level of BAs in blood. However, in our actual operation, we collected blood samples that were residual blood samples in clinical work to reduce the harm of premature infants and comply with medical ethics regulations. On the other hand, we think that results from the real world are more meaningful and conducive to later clinical transformation.

There is a higher incidence rate of NEC in formula-fed preterm infants than in the breastfeeding infants. BAs metabolism was correlated with feeding type in the current study, so we considered whether formula feeding triggered NEC through BAs. In a previous study, apical sodium-dependent bile acids transporter (ASBT) levels, which are responsible for transporting BAs from the apical surface of enterocytes into the cell, were found to be upregulated in NEC. The ileal bile acids binding protein (IBABP), which is responsible for transporting the BAs outside enterocytes, is significantly decreased in NEC [35]. These alterations in BAs transport can lead to the accumulation of BAs in the intestinal epithelial cells in NEC and reduce the number of BAs entering the portal venous circulation, which will further exacerbate the metabolic abnormalities of BAs. Based on these existing reports and our own research experiences, we speculated that increasingly obvious BAs abnormalities may occur after the occurrence of NEC, and this vicious cycle will exacerbate the severity of NEC. We are also completing relevant inference work in other projects.

## Conclusions

BAs metabolism was correlated with the status at birth and after birth in preterm infants and can be used to detect diseases related to premature infants. The novel findings of this study might provide insights into the primary/secondary BA ratio as a diagnostic biomarker for NEC and surgical NEC. As LC-MS/MS is considered the gold standard for analyzing the BAs profile in serum samples, it will be vital to pediatric surgeons for identifying NEC patients who need early surgical intervention.

## Data Availability

Publicly available datasets were analyzed in this study. All datasets presented in this.
